# The Effect of Light Intensity on the Expression of *Leucoanthocyanidin Reductase* in Grapevine Calluses and Analysis of Its Promoter Activity

**DOI:** 10.3390/genes11101156

**Published:** 2020-09-30

**Authors:** Jing Cheng, Keji Yu, Mingyue Zhang, Ying Shi, Changqing Duan, Jun Wang

**Affiliations:** 1 Center for Viticulture and Enology, College of Food Science and Nutritional Engineering, China Agricultural University, Beijing 100083, China; chengjing@cau.edu.cn (J.C.); yukeji@bjfu.edu.cn (K.Y.); shiy@cau.edu.cn (Y.S.); chqduan@cau.edu.cn (C.D.); 2 Key Laboratory of Viticulture and Enology, Ministry of Agriculture and Rural Affairs, Beijing 100083, China; 3 Beijing Advanced Innovation Center for Tree Breeding by Molecular Design, Beijing Forestry University, Beijing 100083, China; 4 China Meat Research Center, Beijing Academy of Food Sciences, Beijing 100068, China; zhmonicayue@163.com

**Keywords:** flavan-3-ol biosynthesis, flavonoid pathway, grapevine callus, leucoanthocyanidin reductase, light, promoter, regulation

## Abstract

To investigate the effect of light intensity on flavonoid biosynthesis, grapevine calluses were subjected to high light (HL, 250 μmol m^−2^ s^−1^) and dark (0 μmol m^−2^ s^−1^) in comparison to 125 μmol m^−2^ s^−1^ under controlled conditions (NL). The alteration of flavonoid profiles was determined and was integrated with RNA sequencing (RNA-seq)-based transcriptional changes of the flavonoid pathway genes. Results revealed that dark conditions inhibited flavonoid biosynthesis. Increasing light intensity affected flavonoids differently—the concentrations of flavonols and anthocyanins as well as the expressions of corresponding genes were less affected, whereas flavan-3-ol concentrations were predominantly increased, which caused enhanced *trans*-flavan-3-ol concentrations. Moreover, genes encoding leucoanthocyanidin reductase (LAR) exhibited different response patterns to light intensity changes—*VviLAR1* expression increased with an increased light intensity, whereas *VviLAR2* expression was insensitive. We further confirmed that the known transcription factors (TFs) involved in regulating flavan-3-ol biosynthesis utilized *VviLAR1* as a target gene in grapevine calluses. In addition, *VviLAR1* promoter activity was more sensitive to light intensity changes than that of *VviLAR2* as determined using a transgenic Arabidopsis leaf system. These results suggested that light intensity had the most prominent effect on *trans*-flavan-3-ols in grapevine calluses and demonstrated that the two LAR genes had different response patterns to light intensity changes.

## 1. Introduction

**Figure 1:**
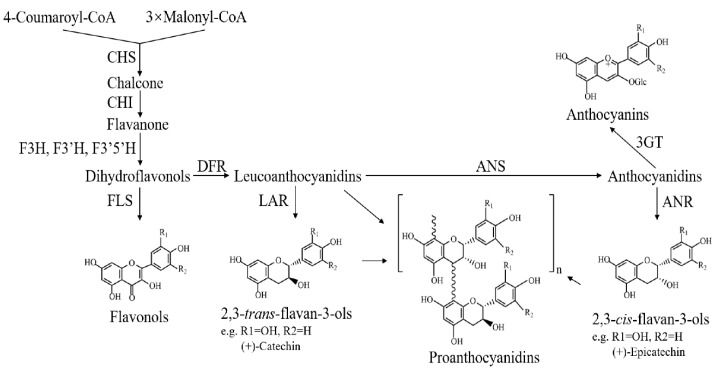
Biosynthetic pathway of the flavonoids of grapes [20]. The enzyme names are abbreviated as follows: CHS, chalcone synthase; CHI, chalcone isomerase; F3H, flavanone 3-hydroxylase; F3′H, flavonoid 3′-hydroxylase; F3′5′H, flavonoid 3′,5′-hydroxylase; FLS, flavonol synthase; DFR, dihydroflavonol 4-reductase; LAR, leucoanthocyanidin reductase; ANS, anthocyanidin synthetase; ANR, anthocyanidin reductase; 3GT, flavonoid-3-*O*-glucosyl transferase. R1 and R2 are H or OH.

Grape (*Vitis vinifera* L.) flavonoids have gained considerable attention as primary determinants of the quality and economic value of grapes and wines [1]. The three major classes of flavonoids found in grapes and wines are proanthocyanidins (PAs), anthocyanins, and flavonols. Of the three classes of flavonoids, PAs are present in the greatest proportion in grapes [2]. PAs, also called condensed tannins, are important flavonoid compounds that give grapes and wines their astringency and bitterness [3]. They are oligomers and polymers of elementary flavan-3-ol units (such as (+)-catechin (C), (−)-epicatechin (EC) and (−)-epicatechin-3-*O*-gallate (ECG)) [4]. Flavan-3-ols are synthesized via the general phenylpropanoid pathway in diverse plant species, which share the same upstream biosynthetic pathway with flavonols and anthocyanins (Figure 1) [3,4,5]. Two enzymes are specific for this branch, namely leucoanthocyanidin reductase (LAR) and anthocyanidin reductase (ANR), which are responsible for the formation of 2,3-*trans*-flavan-3-ols (such as C) and 2,3-*cis*-flavan-3-ols (such as EC, ECG), respectively [3]. In grapes, their function and expression patterns have been well characterized [6,7,8].

The expression of structural genes is regulated by both *cis*- and *trans*-acting elements. *trans*-acting elements, especially transcription factors (TFs), can bind to *cis*-acting elements in the promoter region and consequently regulate gene expression. In grapes, several TFs, including VviMYB5a, VviMYB5b, VviMYBPA1, VviMYBPA2, VviMYBPAR, VviMYBC2-L1 and VviMYBC2-L3, are characterized to involve in regulating the flavan-3-ol biosynthetic branch [9,10,11,12,13,14,15,16]. The overexpression of positive (*VviMYB5a*, *VviMYB5b*, *VviMYBPA1* and *VviMYBPA2*) or negative regulators (*VviMYBC2-L1*) influences the expression of *VviLAR1* and *VviANR*, but not *VviLAR2* [12,13,16]. All the seven TFs mentioned above belong to the R2R3 MYB family. In some situations, bHLH family TFs are also involved in the regulation of flavan-3-ol biosynthesis in grapes. They can interact with R2R3 MYB TFs to form a TF complex, that better modulates target gene expression [11], such as VviMYC1 [17]. *VviMYC1* together with *VviMYBPA1* could activate the promoter of *VviANR,* not *VviLAR1* or *VviLAR2* [17]. Other regulatory mechanisms for (*trans*)-flavan-3-ol genes likely exist. Furthermore, the promoter is a pivotal element for a gene in the regulation network as numerous *cis*-acting elements were present, which can determine the range and the level of gene expression as well as its tissue-, organ-, and developmental stage-specificity in planta [18]. However, the regulation of *VviLAR’s* expression by *cis*-acting elements has rarely been reported.

The biosynthesis of flavonoids is sensitive to the light environment. It is generally thought that sunlight exposure on berries increases the concentrations of flavonoids and elevates the expressions of associated biosynthetic genes [19,20]. Previous research on the effect of light on flavonoid pathways were mostly conducted in the field condition. Sunlight is the light source for field experiments, including UV-light, visible light, and infrared radiation. In addition, the light intensity changes in different areas. For example, Gaotai County, which is located in Gansu Province (West China) has more sunshine than Changli County, which is located in Hebei Province (East China) [21]. Thus, it is necessary to separate the light intensity and light quality (wavelength) when studying the influence of light on flavonoid pathways. Several studies have investigated the effect of light conditions on flavonoid biosynthesis in grapes and found that the range of blue and UV-light showed prominent effects on flavonoids synthesis, especially flavonol and anthocyanin biosynthetic branches [22,23,24,25]. Furthermore, Koyama et al. exhibited that flavan-3-ol synthesis could be induced by visible light [22]. However, research on how light intensity solely affect flavonoid biosynthesis in grapes is still limited.

Plant callus is a convenient model for studying the regulation of plant secondary mechanisms, which enables the controlling of light conditions accurately and independently with other components remaining unchanged. Moreover, the application of a callus system is not restricted to the short growing period of grapes. To date, numerous studies have applied plant calluses, such as grape, apple and haworthia, to investigate the regulation of abiotic factors on secondary metabolic pathways [26,27,28]. Stem tips are generally chosen for callus induction given their strong meristem ability. In addition, grapevine stems have similar compositions of flavonoids with grape berries [29,30].

In this study, different light intensity treatments were performed on grapevine calluses induced from the explants of *Vitis vinifera* L. cv. Cabernet Sauvignon (CS). We then determined the alterations in flavonoid profiles using high-performance liquid chromatography–mass spectrometry (HPLC–MS) and transcriptional levels of the relative pathway genes though RNA sequencing (RNA-seq). Furthermore, in order to investigate why the two LAR genes showed different response patterns to light intensity changes, we also examined the transcriptional level of TFs by RNA-seq and measured the promoter activity of two LAR genes though a transgenic Arabidopsis leaf system.

## 2. Materials and Methods 

### 2.1. Material and Growth Conditions

Friable grapevine calluses were induced from the young stems of CS. The medium for induction and subculture was solid B5 medium with 20 g/L sucrose, 0.25 g/L casein acid hydrolysate, 8.0 g/L agar, 0.2 mg/L α-naphthylacetic acid and 0.4 mg/L kinetin, and adjusted to a pH of 5.8–6.0. The grapevine calluses were placed on tissue culture bottles with three clumps of calluses in one tissue culture bottle. The illumination source came from cool-white fluorescent lamps, which were placed 1 m above the tissue culture bottles. The culture condition for the calluses was 25 ± 1 °C with a light intensity of approximately 125 μmol m^−2^ s^−1^ with a 16/8-h light/dark photocycle. In addition, after measurement, the UV-A emitted by the lamp was a trace amount, whereas UV-B could not be detected. The calluses were subcultured every 21 d.

Pea-sized berries of CS were collected in 2015 on six-year-old vines in the experimental greenhouse at Shangzhuang Experimental Station in Haidian District, Beijing (40°08′12″ N, 116°10′45″ E, altitude 31.3 m). The fresh samples were refrigerated in boxes and taken to the laboratory within a few hours. Then, berries were frozen in liquid nitrogen and stored at −80 °C until further use.

Tobacco (*Nicotiana benthamiana*) and wild-type *Arabidopsis thaliana* (ecotype Col-0) were grown in soil in a growth chamber under cool-white fluorescent lamps (light intensity approximately 125 μmol m^−2^ s^−1^) with a 16/8-h light/dark photocycle at 25 ± 1 °C.

### 2.2. Light Intensity Treatment in Grapevine Callus

Calluses cultured at 25 ± 1 °C under a light/dark photocycle (a 16/8-h light/dark photocycle with light intensity about 125 μmol m^−2^ s^−1^) were placed in climate chambers for 21 d under the following conditions: dark treatment (0 μmol m^−2^ s^−1^), a 16/8-h light/dark photocycle with light intensity about 125 μmol m^−2^ s^−1^ (control, (NL)), and a 16/8-h light/dark photocycle with light intensity approximately 250 μmol m^−2^ s^−1^ (high light, (HL)). The grapevine calluses were immediately collected after treatments for flavonoid extraction and RNA-seq. Three clumps of calluses were placed on one tissue culture bottle, and each treatment used three tissue culture bottles, serving as three independent biological replicates.

### 2.3. Extraction and HPLC–MS Analysis of Flavonoids in Grapevine Callus System

Grapevine calluses were frozen with liquid nitrogen immediately in a precooled mortar and then ground into powder and freeze-dried at −40 °C for 48 h. Subsequently, dried callus powder was placed into the −40 °C refrigerator until further use. Three biological replicates were performed for each set of samples. Flavonols and anthocyanins were extracted according to the previous procedure with a few modifications [31]. Dried callus powder (0.050 g) was macerated in methanol/water mixture (50/50, *v*/*v*) and sonicated at 4 °C for 20 min in darkness. Then, the sample was centrifuged at 4 °C for 5 min (8000 rpm). The supernatant was transferred to a new centrifuge tube and the residue was extracted twice. All the supernatants were pooled and nitrogen-blown at 23 °C. Then, supernatants were dissolved in a methanol/water mixture (50/50, *v*/*v*) and stored at −40 °C. The extraction of PAs followed a previously reported method [32]. Dried callus powder (0.050 g) was mixed with 1.0 mL phloroglucinol buffer (containing 50 g/L phloroglucinol methanol solution, 0.3 M HCl and 5 g/L ascorbic acid) and incubated for 20 min at 50 °C. Then, 1 mL 200 mM aqueous sodium acetate was added to terminate the reaction. The mixture was centrifuged for 15 min (8000 rpm) at 4 °C. The residue was extracted twice. All the supernatants were collected and shaken. After drying with a stream of dry nitrogen at room temperature, the residue was dissolved with a 400 μL methanol/water mixture (50/50, v/v) and stored at −40 °C until detection.

The detection of flavonols in grapevine callus was performed in accordance with the method described by Sun et al. [20]. The method used to analyze anthocyanins in grapevine callus was reported by He et al. [33]. The flavan-3-ol detection method was consistent with that reported by Li et al. [34]. The instruments and configurations involved in the detection of the above components were based on a previous report [35]. Flavonols and anthocyanins were quantified using quercetin-3-*O*-glucoside and malvidin-3-*O*-glucoside as external standards, respectively. Flavan-3-ol concentrations were determined using C, EC, and ECG as external standards. All flavonoid concentrations were expressed as mg/g DW (Dried weight) based on the dry weight of the grapevine callus in this study.

### 2.4. RNA Extraction, cDNA Library Construction and RNA-Seq

Every treatment was assessed in three independent biological replicates, and the samples were sent to Anoroad Co., Ltd. (Beijing, China) for sequencing. Total RNA of grapevine callus was extracted using a Universal Plant Total RNA Extraction Kit (BioTeke, Beijing, China). The RNA sample purity was assessed using a NanoPhotometer^®^ spectrophotometer (Implen, Munich, Germany), RNA concentration was quantified using a Qubit^®^ 3.0 Flurometer (Thermo Fisher, Waltham, MA, USA), and the integrity and concentration of RNA samples were assessed using an Agilent 2100 RNA Nano 6000 Assay Kit (Agilent, Santa Clara, CA, USA). RNA samples were used for the construction of cDNA libraries. Library quality was assessed using the Agilent Bioanalyzer 2100 system (Agilent, USA). Sequencing was performed with Illumina NovaSeq 6000 (Illumina, San Diego, CA, USA) according to the manufacturer’s instructions.

### 2.5. RNA-Seq Data Analysis and Quantitative Real-Time PCR (qRT-PCR) Confirmation

The reads containing adapter, reads containing poly-N (>10%) and low-quality reads (>50% of base with Qphred ≤ 19) were removed from the raw data to obtain clean reads. The V2 version of the grapevine genome was downloaded from http://genomes.cribi.unipd.it/grape/ and used as the reference genome. The reference genome was converted into an index using HISAT2 software and an improved Burrows–Wheeler transform (BWT) algorithm [36]. The gene expression level was estimated based on Fragments per Kilobase per Million Mapped Fragments (FPKMs) [37].

qRT-PCR was performed to confirm the results obtained from RNA-seq analysis. The cDNA was prepared from the same RNA samples applied for RNA-seq analysis using HiScript^®^ II Q RT SuperMix for qPCR (+gDNA wiper) (Vazyme, Nanjing, China). An ABI 7300 Real-Time System (Thermo Fisher, USA) with SYBR qPCR Master Mix (Vazyme, China) was used for qRT-PCR analysis. The specific PCR procedure referred to Sun et al. [38], and data were analyzed as described by Ruijter et al. [39]. *VviUbiquitin1* was used as the reference gene [7]. The gene-specific primers used in this study are provided in Table S1.

### 2.6. Cloning and Sequence Analysis of VviLAR1 Promoter (pVviLAR1) and VviLAR2 Promoter (pVviLAR2)

The region approximately 1.5 kb at the upstream of start codon ATG was regarded as the promoter region, based on the CS genomic sequence (http://genomes.cribi.unipd.it/grape/). Grape genomic DNA was isolated from CS berries using the New Rapid Plant DNA Extraction Kit (BioTeke, China). PCR was performed using specific primer pairs (Table S1), and then PCR products obtained by using Pfu DNA polymerase (TIANGEN, Beijing, China) were cloned into the pMD19-T vector for sequencing validation. The *cis*-acting elements on the p*VviLAR1* and p*VviLAR2* were predicted by the PlantCARE website (http://bioinformatics.psb.ugent.be/webtools/plantcare/html/). Sequence data from this article were deposited at GenBank under accession numbers MT586116 (p*VviLAR1*) and MT586117 (p*VviLAR2*).

### 2.7. Transient Expression in Tobacco Leaves and Stable Expression in Arabidopsis

The promoter of each gene containing restriction sites at its 5′ and 3′ ends was amplified and inserted into binary plasmid pCambia1300-LUC and pCAMBIA1381-GUS to construct transient expression vectors p*VviLAR1*::*LUC* and p*VviLAR2*::*LUC*, as well as stable expression vectors p*VviLAR1*::*GUS* and p*VviLAR2*::*GUS*, respectively. Successfully constructed plant resultant vectors were introduced into *A. tumefaciens* strain GV3101 using the freeze–thaw method.

Then, promoter-*LUC* recombinant Agrobacterium were introduced into the healthy and fully expanded leaves of six-week-old tobacco (*N. benthamiana*). Detailed methods on tobacco infection and LUC detection were described by Sun et al. [38]. The empty vector was used as the negative control in this study.

Promoter-*GUS* recombinant Agrobacterium were introduced into wild-type Arabidopsis though the floral-dipping method [40]. Transgenic Arabidopsis lines were selected on Murashige-Skoog (MS) medium supplemented with 50 mg/L hygromycin B. Transgenesis was confirmed by PCR using promoter-specific primers and GUS staining. The primers used were listed in Table S1. At least three independent lines were selected and used for the further light intensity treatments.

### 2.8. Light Intensity Treatment on Transgenic Arabidopsis

Four-week-old T3 generation transgenic Arabidopsis were placed in the climate chamber for eight hours at 25 °C under the following conditions: high light (light intensity was approximately 250 μmol m^−2^ s^−1^, HL), control condition (light intensity was approximately 125 μmol m^−2^ s^−1^, NL) or dark (0 μmol m^−2^ s^−1^). The leaves were collected immediately after the treatments and were partly subjected to GUS histochemical staining, while the remaining leaves were used to detect the *GUS* transcriptional level by semiquantitative reverse transcription PCR (SqRT-PCR).

The histological GUS staining used in this study followed a previously reported method [41]. Samples were observed under a stereomicroscope and imaged by Charge-coupled device (CCD) after GUS staining. Total RNA was isolated using a MiniBEST Plant RNA Extraction Kit (TaKaRa, Osaka, Japan) and then reverse-transcribed to cDNA using HiScript^®^ II Q RT SuperMix for qPCR (+gDNA wiper) (Vazyme, China). The expression level of *GUS* in transgenic Arabidopsis leaves with light intensity treatments was assessed using SqRT-PCR with specific primers (Table S1). In all cases, PCR conditions were as follows: 94 °C for 1 min, 60 °C for 30 s, and 72 °C for 1 min for 25 cycles. Then, the transcript abundance of the reporter gene *GUS* was analyzed based on the brightness of bands in the agarose gel, and the relative brightness of GUS was normalized to that of *AtActin8* [42].

### 2.9. Statistical Analysis of Data

Data are presented as the mean ± SD (standard deviation). A one-way ANOVA test was conducted using SPSS for Windows version 20.0 (SPSS Inc., Armonk, NY, USA). Pearson’s correlation analysis between the expression of two LAR genes and TFs was performed by using the “psych” package in R statistical environment (3.4.1). The gene co-expression network between structural genes and TFs was created in CytoScape for Windows version 3.7.2 (USA). The column diagram was created in GraphPad Prism 8 (GraphPad Software Inc., San Diego, CA, USA). The line plot was completed using Microsoft Excel 2019 (Microsoft Office, Redmond, WA, USA). The expression profile of structural genes related to flavonoid pathways were visualized with Microsoft Excel 2019 (Microsoft Office, Redmond, WA, USA) and Adobe Illustrator CC2018 (Adobe, San Jose, CA, USA).

## 3. Results

### 3.1. Flavonoid Compositions in Grapevine Calluses under Different Light Intensity Treatments

One of the main goals of the present study was to solely investigate how light intensity regulated flavonoid biosynthesis in grapevine calluses. Two light intensity treatments (dark and HL) were performed on grapevine calluses induced by CS explants, which were cultured under NL. As shown in Figure S1, the light-exposed calluses exhibited a clear red color, whereas calluses cultured in the dark exhibited a simple white color, indicating that light was necessary for anthocyanin production in grapevine calluses. Flavonoid components were further extracted and analyzed from these samples. In total, 24 flavonoid components were detected in grapevine calluses (Table S2), among which one flavonol, six anthocyanins and three flavan-3-ols were quantified.

**Figure 2:**
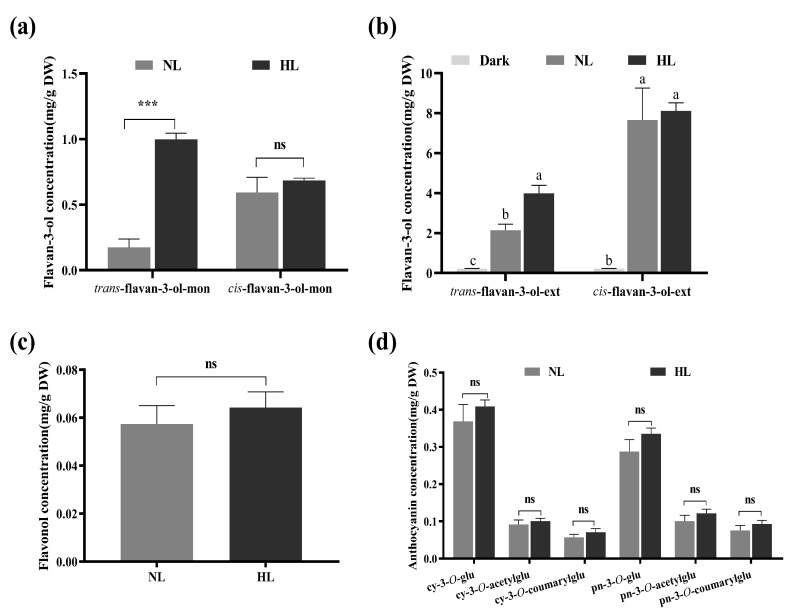
The concentrations of flavonoid components in grapevine calluses under different light intensity treatments. NL and HL mean the grapevine calluses cultured in control condition and high light, respectively. DW: dried weight. (**a**) The concentrations of *cis*/*trans*-flavan-3-ol monomers. “mon” mean monomers, including free monomers and starter units; (**b**) the concentrations of *cis*/*trans*-flavan-3-ols served as extension units in proanthocyanidin (PA) polymerization. “ext” mean extension units. Lower case letters indicate the significant differences among the treatments by one-way ANOVA test (*p* < 0.05); (**c**) the concentration of flavonols; (**d**) the concentration of anthocyanins. cy-3-*O*-glu: cyanidin-3-*O*-glucoside; cy-3-*O*-acetylglu: cyanidin-3-*O*-acetyl-glucoside; cy-3-*O*-coumarylglu: cyanidin-3-*O*-*p*-coumaryl-glucoside; pn-3-*O*-glu: peonidin-3-*O*-glucoside; pn-3-*O*-acetylglu: peonidin-3-*O*-acetyl-glucoside; pn-3-*O*-coumarylglu: peonidin-3-*O*-*p*-coumaryl-glucoside. Each sample was individually assayed in triplicate. Error bars indicate the standard error of the mean. The “ns” or asterisk* in (**a**,**c**,**d**) indicate significant differences between the treatments by one-way ANOVA test (ns, not significant; ***, *p* < 0.001).

The acid catalysis of PAs in the presence of excess phloroglucinol can generate flavan-3-ol monomers and flavan-3-ol conjugates of phloroglucinol, representing PA starter units as well as free monomers and extension units, respectively [43]. Here, from the perspective of constituent units in the PA polymerization process, the concentrations of *trans*/*cis*-flavan-3-ols that served as the monomers and the extension units were calculated (Figure 2a,b). Trace amounts of flavan-3-ol monomers were present in dark calluses. As the light intensity increased, the concentration increased. The concentration of *trans*-flavan-3-ol monomers in the HL group was approximately a five-fold increase when compared with NL ones. However, the concentrations of *cis*-flavan-3-ol monomers derived from the catalysis of ANR (Figure 1) showed no significant difference between HL and NL samples. The pattern of change in the concentration of extension units in grapevine calluses under different light intensity treatments was consistent with that of flavan-3-ol monomers. Furthermore, compared with the dark samples, light-exposed samples accumulated increased amounts of total flavan-3-ols, and maximum level appeared in HL samples, which was about 1.5-fold of that found in NL calluses (Table S3). These findings indicated that the increased concentration of *trans*-flavan-3-ols was responsible for the enhanced level of total flavan-3-ols in grapevine calluses.

Trace amounts of flavonols and anthocyanins were present in the dark samples. Quercetin-3-*O*-glucoside was the only quantifiable flavonol and it was found at similar levels in NL and HL samples (Figure 2c). Cyanidin-3-*O*-glucoside, cyanidin-3-*O*-acetyl-glucoside, cyanidin-3-*O*-*p*-coumaryl-glucoside, peonidin-3-*O*-glucoside, peonidin-3-*O*-acetyl-glucoside, and peonidin-3-*O*-*p*-coumaryl-glucoside were all the products derived from the cyanidin branch pathway. The concentrations of these anthocyanins in HL calluses were slightly increased compared with NL samples (Figure 2d). Moreover, the HL and NL groups showcased similar acylated anthocyanin (acetyl- and coumaroyl-anthocyanins) concentrations (Table S3).

In summary, flavonoid production in grapevine calluses was light-dependent. Enhancing light intensity mainly increased flavan-3-ol concentrations, especially *trans*-flavan-3-ols, whereas to a lesser extent affected flavonol and anthocyanin levels.

### 3.2. Effect of Light Intensity on Flavonoid Pathway Gene Expression

To better understand the transcriptional causation for the alteration in flavonoid profiles in response to light intensity changes in grapevine calluses, total RNAs extracted from the same samples used for flavonoid analysis above were further subjected to RNA-seq.

The parameters used to assess the quality of data obtained from RNA-seq included raw and clean reads numbers, Q30 values, and GC contents. All parameters were within the appropriate ranges (Table S4), suggesting that the RNA-seq data were applicable for further analysis. To validate the expression profiles obtained through RNA-seq, six genes (*VviPAL1*, *VviCHS3*, *VviCHI*, *VviFLS1*, *VviF3H1*, and *VviLAR1*) encoding flavonoid pathway enzymes were selected as targets by performing qRT-PCR in each sample. *VviUbiquitin1* was chosen as the reference gene for normalization [7]. A linear regression analysis of qRT-PCR versus RNA-seq yielded a correlation coefficient value of 0.8124 (Figure S2), meaning that the RNA-seq data were reliable.

**Figure 3:**
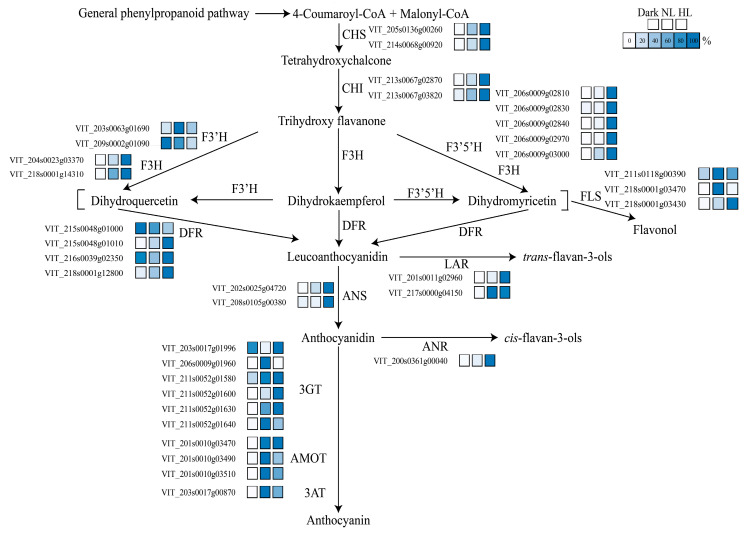
Flavonoid pathway with related transcripts presented in grapevine calluses under different light treatments. NL and HL are the abbreviations of control condition and high light, respectively. The enzyme names are abbreviated as follows: CHS, chalcone synthase; CHI, chalcone isomerase; F3H, flavanone 3-hydroxylase; F3’H, flavonoid 3′-hydroxylase; F3′5′H, flavonoid 3′,5′-hydroxylase; FLS, flavonol synthase; DFR, dihydroflavonol 4-reductase; LAR, leucoanthocyanidin reductase; ANS, anthocyanidin synthetase; ANR, anthocyanidin reductase; 3GT, flavonoid-3-*O*-glucosyl transferase; AMOT, anthocyanin methyltransferase; 3AT, anthocyanin acyltransferase. The colored bars represent the percentage of transcripts for each transcript with 0% being white to 100% being dark blue.

A subset of 13 structural gene families participating in flavonoid metabolism was extracted, and the corresponding expression levels were summarized in Figure 3 and Table S5. The biosynthesis of flavan-3-ols, anthocyanins, and flavonols shares the common early steps of the flavonoid pathway, and the enzymes involved in the upstream pathway are chalcone synthase (CHS) and chalcone isomerase (CHI) [44]. Genes encoding CHS and CHI responded similarly to light intensity changes—their expressions were activated by the dark and transcript levels were up-regulated as the light intensity increased.

The hydroxylation of flavonoids is mediated by the enzyme activity of flavonoid 3′-hydroxylase (F3’H) and flavonoid 3′,5′-hydroxylase (F3′5′H), which catalyze the hydroxylation of naringenin and dihydrokaempferol at the 3′ and 3′5′ positions of the B-ring, respectively [45]. *F3’H* family genes were expressed in all treatments, but the transcriptional levels were slightly decreased in the HL group. Conversely, the expressions of *F3*′5′*H* family genes were difficult to induce by light. These findings indicate that the F3’H branch could be activated more easily than the F3′5′H pathway, consequently promoting the production of F3′H pathway-derived metabolites in grapevine calluses. In addition, the upstream enzyme CHI can interact directly with F3’H to form an enzyme complex, thereby accelerating the compound exchange between adjacent enzyme members and improving the catalytic efficiency of the entire pathway [46,47].

Flavonol synthase (FLS) and dihydroflavonol 4-reductase (DFR) compete for dihydroflavonols [44]. FLS is an enzyme that is exclusively responsible for flavonol production. Here, *VviFLS*s exhibited different response patterns to light intensity changes. *VviFLS4* (VIT_218s0001g03470) was largely expressed merely in the NL group. As the light intensity increased, the expression of *VviFLS5* (VIT_218s0001g03430) elevated, whereas VIT_211s0118g00390 maintained a low transcriptional level. DFR is the first of the “late” enzymes of the flavonoid pathway and converts dihydroflavonols into leucoanthocyanidins [44]. *VviDFRs* were expressed in all light treatments, and the maximum transcriptional level of the predominant gene (VIT_218s0001g12800) occurred in the HL group. In addition, the overall transcriptional levels of *VviDFRs* were considerably increased compared with *VviFLSs* in all samples. Hence, DFR could compete with more substrates to direct flavonoid biosynthesis towards the production of anthocyanins and flavan-3-ols.

LAR and ANR are two key enzymes that are involved in flavan-3-ol biosynthesis as they convert leucoanthocyanidins and anthocyanidins, respectively, into the corresponding 2,3-*trans/cis* flavan-3-ols (such as C/EC) [44]. *VviLAR1* (VIT_201s0011g02960) and *VviLAR2* (VIT_217s0000g04150) encode LAR [7]. In this study, the expression patterns of the two genes completely differed in response to light intensity change. The transcriptional level of *VviLAR1* was maximal in HL calluses, followed by NL calluses, which was 10% of that in HL calluses. *VviLAR2* expression was enhanced in light-exposed calluses, but expression was still low, indicating that the capacity of light to induce *VviLAR2* expression was limited. The response pattern of *VviANR* (VIT_200s0361g00040) to different light intensities was consistent with that of *VviLAR1*. Combined with the changes of flavan-3-ol concentrations in grapevine calluses under different light intensity conditions (Figure 2a,b and Table S3), these results suggested that *VviLAR1* played an important role in flavan-3-ol production in grapevine calluses during light intensity changes.

Anthocyanidin synthetase (ANS) is a pivotal enzyme for both anthocyanin formation and flavan-3-ol biosynthesis. ANS converts leucoanthocyanidins into unstable anthocyanidins [44]. The transcriptional level of one *ANS* gene (VIT_202s0025g04720) was positively correlated with light intensity changes and the transcriptional level in HL calluses was approximately four-fold of that in NL levels. However, another *ANS* family gene (VIT_208s0105g00380) was hardly expressed in all calluses. Flavonoid-3-*O*-glucosyl transferases (3GTs) are a group of enzymes that catalyze anthocyanidin glycosylation at the C3 position of the C-ring, leading to the absolute branch pathway of anthocyanin synthesis [44]. Subsequently, the glycosyl-moiety of anthocyanins could be further modified via anthocyanin methyltransferase (AOMT) and anthocyanin acyltransferase (3AT) activities [48]. The expression of anthocyanin-specific genes was found to require light stimulation. The expression of a portion of *3GT* family genes was activated by NL but inhibited by HL, whereas others were exclusively activated by HL. *VviAOMT1*, *VviAOMT2* and *Vvi3AT* exhibited maximum expression levels in NL calluses, but their expression was inhibited by HL. Taken together, the majority of transcripts specific for anthocyanin biosynthesis were light-dependent, but their response patterns to light intensity changes exhibited differences.

### 3.3. Screening for Potential TFs to Regulate the Expressions of VviLAR1 and VviLAR2

Based on the above results, we found that light significantly enhanced the *trans*-flavan-3-ols concentration and the transcriptional level of *VviLAR1* in grapevine calluses. However, the expression of another LAR gene, *VviLAR2*, was not sensitive to light intensity changes. These findings indicated that the two LAR genes were regulated differently. It is well known that *cis*- and *trans*-acting elements are largely responsible for regulating gene transcription in the plant kingdom. Hence, to clarify the potential mechanism that determined the different expression patterns of the two *VviLARs* in response to light intensity changes, we further explored this from the perspective of regulation of both *trans*- and *cis*-acting elements.

The expression levels of all TFs were extracted from RNA-seq data, and TFs with very low expression abundance in all treatments (FPKM < 1) were filtered out from this analysis. Correlations between the expression of TFs and *VviLAR1* as well as *VviLAR2* were calculated. TF with a strong correlation (Pearson correlation coefficient |PCC| > 0.8 and *p*-value < 0.01) with at least one of them was considered to potentially regulate the expression of *VviLAR1* and/or *VviLAR2*. A total of 171 TFs were screened out (Tables S6 and S7), only 11 TFs exhibited strong correlations with both LAR genes, including bHLH130 (VIT_206s0004g01740), bHLH111 (VIT_213s0019g00540), bHLH94 (VIT_214s0128g00110), UNE10 (VIT_215s0021g02690), E2FC (VIT_218s0001g14110), RAP2-7 (VIT_206s0004g03590), HSEB-3 (VIT_208s0007g08750), MADS18 (VIT_218s0001g09540), ETC3 (VIT_212s0059g02360), TCP2 (VIT_210s0003g03910), and ASIL1 (VIT_211s0016g04130).

**Figure 4:**
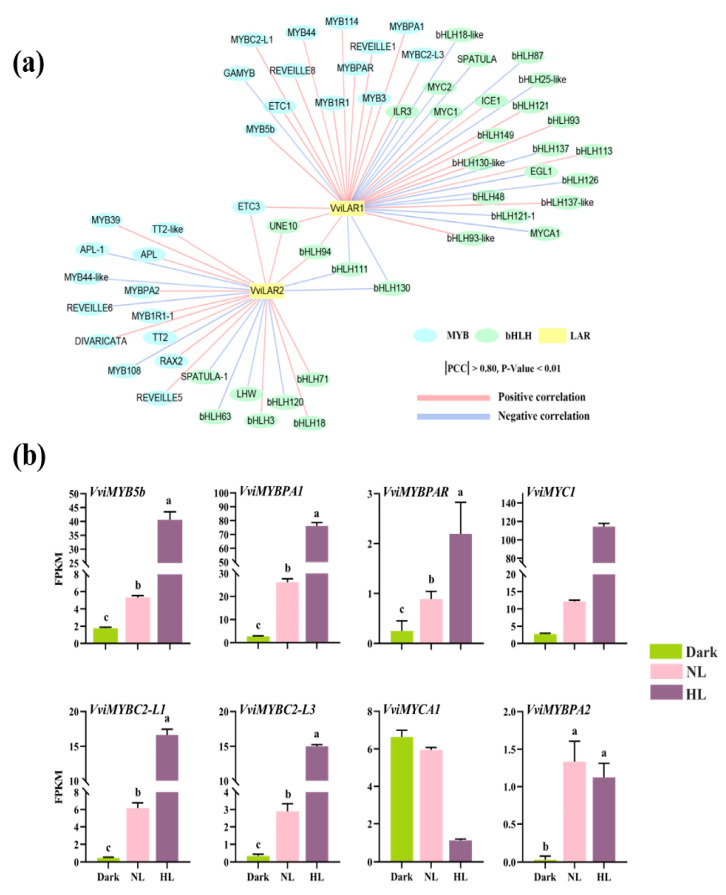
Screening for transcription factors (TFs) regulating two LAR genes’ expression in grapevine calluses. (**a**) Gene co-expression network between *VviLARs* and TFs. Structural genes and TFs are represented by square and oval, respectively. Lines in light red and light blue represent positive or negative associations between LAR genes and TFs (|PCC| > 0.8; *p* < 0.01). (**b**) The expressions of known TFs regulate *trans*-flavan-3-ol biosynthesis in grapevine calluses under different light intensity treatments. HL: grapevine calluses treated in high light; NL: grapevine calluses cultured in control condition. The expression level of TFs was estimated based on Fragments per Kilobase per Million Mapped Fragments (FPKM). Data are expressed as means ± SD of three biological replicates. Lower case letters indicated significant differences analyzed by one-way ANOVA tests.

MYB and bHLH TFs are key modulators of plant metabolism and development [11]. *trans*-flavan-3-ol biosynthesis is driven by R2R3 MYB TFs and, in some cases, interactions with bHLH proteins to form TF complex in grapes [11]. Here, emphasis was given to the TFs that belonged to the MYB and bHLH families. TFs that belonged to MYB (27 genes) and bHLH (32 genes) families with strong correlations were determined and are shown in Figure 4a. Six TFs that have been previously identified to be involved in the regulation of the *trans*-flavan-3-ol biosynthetic branch (VviMYB5b, VviMYBPA1, VviMYBPA2, VviMYBPAR, VviMYBC2-L1, and VviMYBC2-L3) were also screened out. The expression levels of five TFs, including *VviMYB5b*, *VviMYBPA1*, *VviMYBPAR*, *VviMYBC2-L1*, and *VviMYBC2-L3*, were positively correlated with that of *VviLAR1*, and the PCC values were 0.9979, 0.9713, 0.9071, 0.9589, and 0.9954, respectively. The expression of these genes was enhanced as light intensity increased (Figure 4b), indicating that these genes were strongly modulated by light. Compared with the other six TFs, *VviMYBPAR* was always expressed at a low level. Only the expression of *VviMYBPA2* was strongly correlated with that of *VviLAR2* (PCC = 0.9592). Trace *VviMYBPA2* expression was noted in all light intensity conditions (Figure 4b). Our findings indicated that in *trans*-flavan-3-ol biosynthesis of grapevine calluses, *VviLAR1* was the target gene of VviMYB5b, VviMYBPA1, VviMYBPAR, VviMYBC2-L1, and VviMYBC2-L3, whereas *VviLAR2* was the target gene for VviMYBPA2.

In addition, we noted that VviMYC1, which could physically interact with VviMYBPA1 to activate the *VviANR* promoter [17], had also been shown to positively regulate *VviLAR1*. The expression of *VviMYC1* was positively correlated with the light intensity (Figure 4b). VviMYCA1, a bHLH family TF that modulates *VviANR* and *VviUFGT* [49], also potentially regulated *VviLAR1* with its expression level being inversely related to light intensity (Figure 4b).

Other screened TFs, including members of WRKY, ERF, MADS, and bZIP families, exhibited strong correlations with *VviLAR1* and/or *VviLAR2*, suggesting that these TFs might also be implicated in the regulation of the *trans*-flavan-3-ol biosynthetic branch, but still need to be proven experimentally.

### 3.4. pVviLAR1 Activity Was More Sensitive than pVviLAR2 Activity in Response to Light Intensity Changes

**Figure 5:**
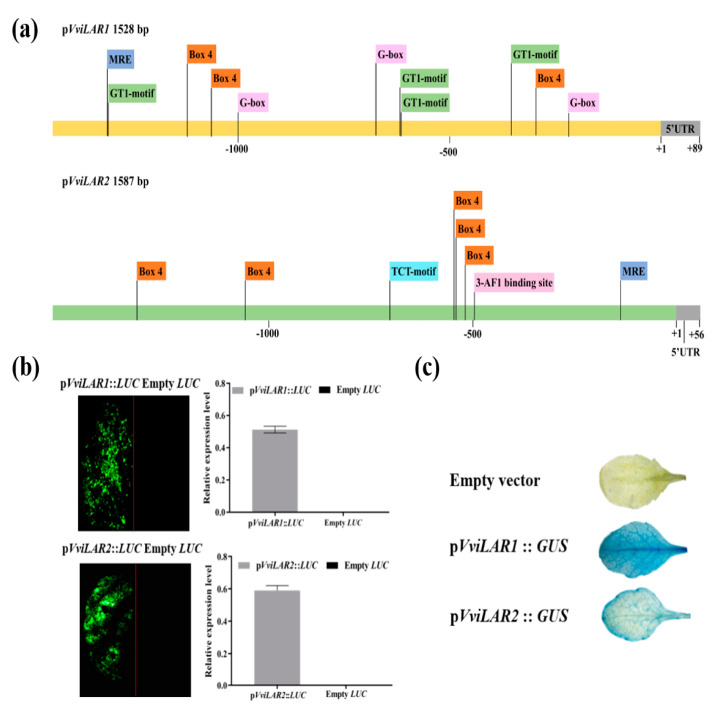
Sequence analysis and examination of the cloned promoter activity. (**a**) The putative *cis*-acting elements in both orientations of the p*VviLAR1* and p*VviLAR2* predicted by the PlantCARE website, only light-responsive elements (LREs) are shown; (**b**) tobacco leaves were transformed with equal amount of p*VviLARs*::*LUC* construct (left side) and Empty *LUC* construct (right side), respectively. Left panel: fluorescence imaging. Right panel: relative expression of *LUC* estimated by image J software; (**c**) GUS staining on the expression of *GUS* driven by promoters in the Arabidopsis leaves.

To assess the response of p*VviLAR1* and p*VviLAR2* to light intensity changes, we further cloned the promoters of these two *VviLARs* from the genomic DNA of CS berries. The sizes of p*VviLAR1* and p*VviLAR2* were 1528 and 1587 bp, respectively (Figure S3). The sequence of p*VviLAR1* exhibited a 99% identity with that of *V. vinifera* L cv. Shiraz, for which the transcription start site (TSS) has been reported as thymine at 88 bp from 5’ upstream of the ATG [12]. The TSS of p*VviLAR2* was predicted to be the cytosine at 56 bp from 5’ upstream of the ATG using Softberry TSSP (http://linux1.softberry.com/). Moreover, the nucleotide sequences of p*VviLAR1* and p*VviLAR2* were not similar, which was consistent with a previous study [50]. In both promoter regions, the core elements TATA-box and CAAT-box necessary for transcription were existed (Table S8). In addition, putative environmental stress-response and plant hormone-response *cis*-acting elements as well as binding sites for TFs were also found (Table S8), indicating that *VviLAR1* and *VviLAR2* might participate in multiple abiotic response metabolisms in grapes. Among the *cis*-acting elements existing in promoter regions, light-responsive elements (LREs) represented the largest group with different types, numbers and arrangement (Figure 5a). Four types of LREs (GT1-motif, Box 4, G-Box, and MRE) existed in p*VviLAR1* that appeared 11 times in total, and except MRE, each of the other three types occurred at least thrice. Four types of LREs (Box 4, MRE, TCT-motif, and 3-AF1-binding site) were also found in the region of p*VviLAR2*, but the total number was three less than that of p*VviLAR1*. In addition, only Box 4 appeared repeatedly. How these LREs collectively influence *VviLARs’* expression response to light intensity changes remains unknown to date.

Then, to assess whether the p*VviLAR1* and p*VviLAR2* we cloned were functional, the two promoter-*LUC* transient expression constructs were transformed into tobacco leaves. After applying D-luciferin, clear fluorescence signals were detected in the leaf portion infected by *A. tumefaciens* strains with the constructs of p*VviLAR1*::*LUC* or p*VviLAR2*::*LUC*, whereas fluorescence was absent in the control area infiltrated with *A. tumefaciens* solution carrying empty vectors (Figure 5b). The results revealed that these two cloned promoters were both functional in planta.

**Figure 6:**
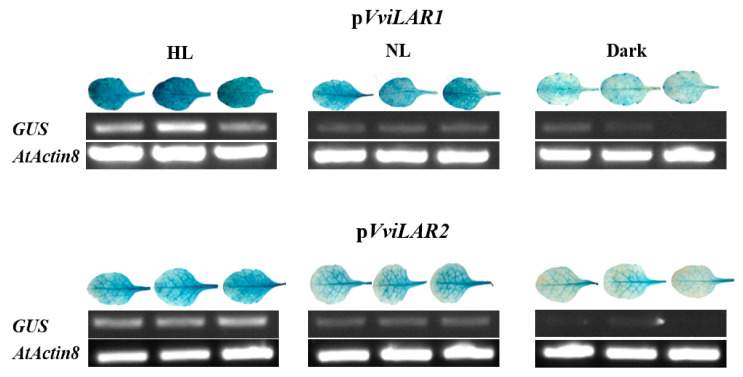
Histochemical GUS staining and expression levels of reporter gene *GUS* in transgenic Arabidopsis leaves with promoter-*GUS* fusion vectors under different light intensity treatments. HL: the Arabidopsis treated under high light. NL: the Arabidopsis treated under control condition. In each treatment, the left, middle, and right leaf came from different lines of transgenic Arabidopsis carrying p*VviLAR1::GUS* or p*VviLAR2::GUS*, respectively. *AtActin8* was used as a control for the semiquantitative reverse transcription PCR (SqRT-PCR). Amplified products were electrophoresed on 1.5%(*w*/*v*) agarose gels.

Then, we used a transgenic Arabidopsis leaf system to test the response patterns of p*VviLAR1* and p*VviLAR2* to different light intensities. We fused *GUS* with p*VviLAR1* or p*VviLAR2* on binary vectors and transferred the two constructs into wild-type Arabidopsis. Histochemical GUS staining showed that both p*VviLAR1*::*GUS* and p*VviLAR2*::*GUS* were functional in the leaves of the transgenic lines (Figure 5c).

p*VviLAR1*::*GUS* or p*VviLAR2*::*GUS* transgenic Arabidopsis seedlings were first grown in a greenhouse for four weeks and then subjected to the treatments of dark and HL, separately. Leaves were immediately sampled after the treatments and then subjected to GUS staining and SqRT-PCR detection. For both p*VviLAR1*::*GUS* and p*VviLAR2*::*GUS* lines, the GUS staining assay results showed that the dark treatment resulted in the weakest GUS activity, whereas the HL treatment exhibited the strongest activity (Figure 6). The results were consistent with the transcript abundance of *GUS* (Figure 6 and Figure S4). These findings demonstrated that the abundance of the *GUS* transcript level increased as the light intensity increased, which further supports the results obtained in GUS staining assays. These results indicated that both promoters could be induced by light. During light intensity change, the *GUS* expression level induced by p*VviLAR1* was more sensitive than that of p*VviLAR2*, suggesting light could more easily enhance the activity of p*VviLAR1* instead of p*VviLAR2*, which may explain the different responses of the two *VviLARs* to light intensity change.

## 4. Discussion

### 4.1. Light Intensity Mainly Regulated trans-Flavan-3-ol Biosynthesis in Grapevine Calluses

In our study, flavonol, anthocyanin and flavan-3-ol biosynthesis were light-dependent—this finding was consistent with previous literature [2,22]. However, enhancing light intensity in light-exposed grapevine calluses mainly induced flavan-3-ol production, especially *trans*-flavan-3-ols, not flavonols and anthocyanins. In the field condition, the light intensity level of the grape bunches without leaf removing or shading is between 100 and 400 μmol m^−2^ s^−1^ [19]. The results we obtained provide insights for studying the mechanism of coloration and PA biosynthesis in grape berries grown in the nature state in different areas. Three factors may explain the insignificant differences in flavonol concentrations between the NL and HL groups. First, all of the known *VviFLSs* can control flavonol production in grapes together [51]. *VviFLS4* was highly expressed in NL samples and *VviFLS5* exhibited the maximal expression in the HL group, thus ensuring flavonol production under NL and HL conditions, respectively. Second, flavonol levels were affected by the direct competition between FLS and DFR [52]. The expression of *VviDFRs* was enhanced by HL, which could compete with more substrates to produce anthocyanins and flavan-3-ols. Third, flavonol production is largely stimulated by UV-light, not visible light [22,53]. The source of illumination used in the present study was fluorescent tubes, and the wavelength range was 400–750 nm, which belonged to visible light. This feature may result in the limited production of flavonols. The levels of anthocyanins were not modified by increasing light intensity in light-exposed calluses. We postulate that similar to *VviFLSs*, all these anthocyanin genes contribute to the formation of anthocyanins. Moreover, a high light intensity can accelerate both the chemical and enzymatic degradation of anthocyanins [54,55]. Furthermore, in our study, the light intensity in the HL treatment was relative to the dark condition. A previous study has reported that the cell suspension culture originated from Gamy Red grape berry could accumulate more cyanidin-form anthocyanins when the cell suspension culture was used to harness metabolic response to excess high light (2500 μmol m^−2^ s^−1^) [56]. Therefore, it is possible that the light intensity used in the present study has not yet reached the threshold for the response of grapevine cells to anthocyanin biosynthesis.

Our data demonstrated that the total flavan-3-ol concentration was highly correlated with *trans*-flavan-3-ol production in grapevine calluses during light intensity changes. *VviLAR1* and *VviLAR2* are two genes encoding LAR with 60% identity at the protein level in Shiraz [7]. The expression level of *VviLAR1* was positively correlated with light intensity, which might guarantee sufficient LAR activities to compete with more substrates to produce *trans*-flavan-3-ols in grapevine calluses under HL. Conversely, the expression of *VviLAR2* was unaffected by light intensity. Our findings on alternations in the transcription levels of the two LAR-specific genes to light intensity changes were consistent with the results obtained by Koyama et al. [22]. In addition, the concentration of *cis*-flavan-3-ols was slightly increased in HL calluses compared with NL calluses. *VviANR* is a key gene involved in the regulation of flavan-3-ol biosynthesis [57], and its expression level increased with light intensity. One possible scenario is that the enzymatic amount and activity of ANR already reached a saturated state in NL calluses, and the further increase in *VviANR* expression might not result in a significant increase in *cis*-flavan-3-ol production. Additionally, the substrate channeling of the flavonoid compounds between DFR and LAR was established using molecular modelling, and the formation of the DFR–LAR complex accelerated the DFR product towards the LAR cavity [58]. Furthermore, ANS is an enzyme that directly competed with ANR for anthocyanidins, and the expression levels of *VviANSs* were elevated with the light intensity, and thus would obtain substrates for anthocyanin metabolism. 

### 4.2. Two LAR Genes Processed Different Response Patterns to Light Intensity in Grapevine Calluses

*VviLAR1* and *VviLAR2* have different expression patterns among organs and developmental stages in grapes [7], indicating different regulations of these two genes. Here, the two *VviLARs* also exhibited different response patterns to light intensity changes. Gene expression is regulated by both *cis*- and *trans*-acting elements. The characterization of LREs existing in the promoter region has been widely used as a convenient starting point for understanding the light control of gene expression [59]. Both p*VviLAR1* and p*VviLAR2* were light-inducible, but p*VviLAR1* was more sensitive to light intensity changes than p*VviLAR2*. We postulated that the relative further distance between the core TATA box (generally located around −30 bp to −50 bp upstream of the TSS) and TSS in p*VviLAR2* compared with that of p*VviLAR1* may account for the weaker activity since the difference in the position affects the binding affinity of TFIID (a TATA element-binding protein) with the TATA box [60]. Promoters of the two genes showed that respective sequences processed several LREs, but the type, number and spatial distribution were distinctly different. The total number of LREs in p*VviLAR1* was greater than that of p*VviLAR2*. Three types of LREs in p*VviLAR1* appeared at least thrice, while only one LRE was repeatedly present in p*VviLAR2*. Moreover, the LREs in p*VviLAR1* were evenly spaced, whereas LREs in p*VviLAR2* were concentrated between −750 and +1 bp. It has been noted that the occurrence frequency of LREs in the promoter region could influence the regulatory strength [44]. For example, the tobacco rbcMT-T promoter contained seven GT1 motifs. The promoter drove *GUS* expression even in the dark if all the seven GT1 motifs were present, or only drove the expression in the light when only two GT1 motifs were present [61]. In addition, an array of MYB and MYC recognition elements were existed in p*VviLAR1* and p*VviLAR2*. Several well-identified TFs preferred *VviLAR1* as the target gene, and consequently regulated its expression in grapevine calluses. VviMYBC2-L1 and VviMYBC2-L3 are transcriptional repressors in flavan-3-ol biosynthesis [9,10], and their gene expression levels were enhanced as light intensity increased in grapevine calluses. Actually, for specific regulatory mechanisms, flavan-3-ol-synthesis genes may require different amounts of *VvMYBC2-L1* transcripts [9]. For example, in grape seeds, the low-level transcripts were sufficient to control *VviLAR1* expression, whereas a large amount was required for *VviLDOX* [9]. Hence, we believe that the suppression of *VviLAR1* expression in grapevine calluses might require large amounts of negative regulators. In general, the sensitivity of p*VviLAR1* shows toward light intensity changes and the known TFs involved in regulating flavan-3-ol biosynthesis preferring to regulate *VviLAR1* expression represent a potential mechanism that determines the different response patterns of two LAR genes in grapevine calluses. In p*VviLAR2*, there were still other stress-responsive elements that existed, such as drought-responsive elements (MBS element), hormone-responsive elements (CARE motif, P-box), and elements responding to oxidative stress (GC-motif), indicating that a drought, gibberellin or oxidative environment might be involved in regulating the expression of *VviLAR2*. Furthermore, a majority of TFs also showed the potential to regulate the expression of the two LAR genes, but their functions need to be systematically characterized. 

## 5. Conclusions

Our findings demonstrated that flavonol, anthocyanin and flavan-3-ol biosynthesis required light stimulation in grapevine calluses. Enhancing light intensity only positively affected flavan-3-ol metabolism—especially *trans*-flavan-3-ol biosynthesis. Two structural genes exclusive to *trans*-flavan-3-ol biosynthesis exhibited different response patterns to light intensity changes in grapevine calluses. The transcriptional level of *VviLAR1* increased as the light intensity increased, whereas *VviLAR2* was not sensitive. Further, our evidence corroborated that both *cis*- and *trans*-acting elements played pivotal roles in regulating the expression of the two LAR genes. VviMYB5b, VviMYBPA1, VviMYBPAR, VviMYBC2-L1, and VviMYBC2-L3 utilized *VviLAR1* as the target gene in grapevine calluses. In addition, the promoter activity of *VviLAR1* was more sensitive than that of *VviLAR2* to light intensity changes as demonstrated using the transgenic Arabidopsis leaf system. Our study suggested that light intensity had the most prominent effect on *trans*-flavan-3-ols in grapevine calluses and the two LAR genes expressed themselves differently in response to light intensity changes.

## Supplementary Materials

The following are available online at https://www.mdpi.com/2073-4425/11/10/1156/s1, Figure S1: Phenotypes of grapevine calluses treated with different light intensities. NL, control condition; HL, high light; Figure S2: Comparison of gene expression determined by RNA-Seq and by qRT-PCR. Data were from six genes across three different light intensity treatments, *VviUbiquitin1* was used as the reference gene in qRT-PCR. The RNA-Seq values (normalized) were plotted against the qRT-PCR values (normalized). The normalized values were determined by calculating the relative expression ratio value for each treatment relative to the highest value for each gene. Linear regression analysis gave an overall coefficient of variation of 0.8124; Figure S3: PCR products of p*VviLAR1* and p*VviLAR2* analyzed with 1.5% agarose gel electrophoresis. (a) Lane 1: p*VviLAR1*, lane M1: DNA marker IV (TIANGEN, China); (b) The original gel of the agarose gel in Figure S3a; (c) Lane 2: p*VviLAR2*, lane M2: DNA marker D2000 (TIANGEN, China); (d) The original gel of the agarose gel in Figure S3c; Figure S4: The original gel of GUS and *AtActin8* expression detection in Figure 6. M: DNA marker D2000 (TIANGEN, China); HL: high light; NL: control condition. The number “1–3” means the different lines used for the light intensity treatments, which corresponded to the left, middle, and right leaf of p*VviLAR1* or p*VviLAR2* in each light intensity treatment in Figure 6, respectively. Table S1: The primers used in this study. The sequence shown in boldface represents the restriction sites, the sequence underlined is the protective bases; Table S2: Flavonoid components detected in grapevine calluses under different light intensity treatments; Table S3: Flavonoids in the grapevine calluses under different light intensity treatments; Table S4: Summary of RNA-Seq, assembly and sequencing reads mapping to the reference genome; Table S5: The expression levels of flavonoid pathway with related transcripts presented in grapevine calluses under different light intensity treatments; Table S6: A list of the expression of TFs strongly correlated with the expression of *VviLAR1* in grapevine calluses under light intensity treatments (|PCC| > 0.80, *p* < 0.01); Table S7: A list of the expression of TFs strongly correlated with the expression of *VviLAR2* in grapevine calluses under light intensity treatments (|PCC| > 0.80, *p* < 0.01); Table S8: *cis*-Acting elements presented in p*VviLAR1* and p*VviLAR2*.

## References

[1] Adams D.O. (2006). Phenolics and ripening in grape berries. Am. J. Enol. Vitic..

[2] Downey M.O., Harvey J.S., Robinson S.P. (2008). The effect of bunch shading on berry development and flavonoid accumulation in Shiraz grapes. Aust. J. Grape Wine Res..

[3] Dixon R.A., Xie D.Y., Sharma S.B. (2005). Proanthocyanidins—A final frontier in flavonoid research?. New Phytol..

[4] Xie Y.D., Dixon R.A. (2005). Proanthocyanidin biosynthesis—Still more questions than answers?. Phytochemistry.

[5] Ferrer J.L., Austin M.B., Stewart C., Noe J.P. (2008). Structure and function of enzymes involved in the biosynthesis of phenylpropanoids. Plant Physiol. Biochem..

[6] Xie D.Y., Sharma S.B., Paiva N.L., Ferreira D., Dixon R.A. (2003). Role of anthocyanidin reductase, encoded by BANYULS in plant flavonoid biosynthesis. Science.

[7] Bogs J., Downey M.O., Harvey J.S., Ashton A.R., Tanner G.J., Robinson S.P. (2005). Proanthocyanidin synthesis and expression of genes encoding leucoanthocyanidin reductase and anthocyanidin reductase in developing grape berries and grapevine leaves. Plant Physiol..

[8] Yu K., Jun J.H., Duan C., Dixon R.A. (2019). VvLAR1 and VvLAR2 are bifunctional enzymes for proanthocyanidin biosynthesis in grapevine. Plant Physiol..

[9] Huang Y.F., Vialet S., Guiraud J.L., Torregrosa L., Bertrand Y., Cheynier V., This P., Terrier N. (2014). A negative MYB regulator of proanthocyanidin accumulation, identified through expression quantitative locus mapping in the grape berry. New Phytol..

[10] Cavallini E., Matus J.T., Finezzo L., Zenoni S., Loyola R., Guzzo F., Schlechter R., Ageorges A., Arce-Johnson P., Tornielli G.B. (2015). The phenylpropanoid pathway is controlled at different branches by a set of R2R3-MYB C2 repressors in grapevine. Plant Physiol..

[11] Hichri I., Barrieu F., Bogs J., Kappel C., Delrot S., Lauvergeat V. (2011). Recent advances in the transcriptional regulation of the flavonoid biosynthetic pathway. J. Exp. Bot..

[12] Bogs J., Jaffé F.W., Takos A.M., Walker A.R., Robinson S.P. (2007). The grapevine transcription factor VvMYBPA1 regulates proanthocyanidin synthesis during fruit development. Plant Physiol..

[13] Terrier N., Torregrosa L., Ageorges A., Vialet S., Verries C., Cheynier V., Romieu C. (2009). Ectopic expression of VvMYBPA2 promotes proanthocyanidin biosynthesis in grapevine and suggests additional targets in the pathway. Plant Physiol..

[14] Koyama K., Numata M., Nakajima I., Gotoyamamoto N., Matsumura H., Tanaka N. (2014). Functional characterization of a new grapevine MYB transcription factor and regulation of proanthocyanidin biosynthesis in grapes. J. Exp. Bot..

[15] Deluc L., Barrieu F., Marchive C., Lauvergeat V., Decendit A., Richard T., Carde J.P., Merillon J.M., Hamdi S. (2006). Characterization of a grapevine R2R3-MYB transcription factor that regulates the phenylpropanoid pathway. Plant Physiol..

[16] Deluc L., Bogs J., Walker A.R., Ferrier T., Decendit A., Merillon J.M., Robinson S.P., Barrieu F. (2008). The transcription factor VvMYB5b contributes to the regulation of anthocyanin and proanthocyanidin biosynthesis in developing grape berries. Plant Physiol..

[17] Hichri I., Heppel S.C., Pillet J., Léon C., Czemmel S., Delrot S., Lauvergeat V., Bogs J. (2010). The basic helix-loop-helix transcription factor MYC1 is involved in the regulation of the flavonoid biosynthesis pathway in grapevine. Mol. Plant.

[18] Liu Z., Luo Q.H., Wang J.M., Li X.F., Yang Y. (2015). Functional characterization and analysis of the Arabidopsis UGT71C5 promoter region. Genet. Mol. Res..

[19] Matus J.T., Loyola R., Vega A., Pena-Neira A., Bordeu E., Arce-Johnson P., Alcalde J.A. (2009). Post-veraison sunlight exposure induces myb-mediated transcriptional regulation of anthocyanin and flavonol synthesis in berry skins of *Vitis vinifera*. J. Exp. Bot..

[20] Sun R.Z., Cheng G., Li Q., He Y.N., Wang Y., Lan Y.B., Li S.Y., Zhu Y.R., Song W.F., Zhang X. (2017). Light-induced variation in phenolic compounds in Cabernet Sauvignon grapes (*Vitis vinifera* L.) involves extensive transcriptome reprogramming of biosynthetic enzymes, transcription factors, and phytohormonal regulators. Front. Plant Sci..

[21] Sun R.Z., He F., Lan Y.B., Xing R.R., Liu R., Pan Q.H., Wang J., Duan C.Q. (2015). Transcriptome comparison of Cabernet Sauvignon grape berries from two regions with distinct climate. J. Plant Physiol..

[22] Koyama K., Ikeda H., Poudel P.R., Goto-Yamamoto N. (2012). Light quality affects flavonoid biosynthesis in young berries of Cabernet Sauvignon grape. Phytochemistry.

[23] Zoratti L., Karppinen K., Escobar A.L., Häggman H., Jaakola L. (2014). Light-controlled flavonoid biosynthesis in fruits. Front. Plant. Sci..

[24] Liu L.L., Gregan S., Winefield C., Jordan B. (2015). From UVR8 to flavonol synthase: UV-B-induced gene expression in Sauvignon blanc grape berry. Plant Cell Environ..

[25] Martinez-Luescher J., Torres N., Hilbert G., Richard T., Sanchez-Diaz M., Delrot S., Aguirreolea J., Pascual I., Gomes E. (2014). Ultraviolet-B radiation modifies the quantitative and qualitative profile of flavonoids and amino acids in grape berries. Phytochemistry.

[26] Cetin E. (2014). Induction of secondary metabolite production by UV-C radiation in *Vitis vinifera* L. Öküzgözü callus cultures. Biol. Res..

[27] Sun J., Wang Y., Chen X., Gong X., Wang N., Ma L., Qiu Y., Wang Y., Feng S. (2017). Effects of methyl jasmonate and abscisic acid on anthocyanin biosynthesis in callus cultures of red-fleshed apple (*Malus sieversii* F. *niedzwetzkyana*). Plant Cell Tiss. Org..

[28] Chen Y.M., Huang J.Z., Hou T.W., Pan I.C. (2019). Effects of light intensity and plant growth regulators on callus proliferation and shoot regeneration in the ornamental succulent Haworthia. Bot. Stud..

[29] Jordao A.M., Ricardo-da-Silva J.M., Laureano O. (2001). Evolution of catechins and oligomeric procyanidins during grape maturation of Castelao Frances and Touriga Francesa. Am. J. Enol. Vitic..

[30] Souquet J.M., Labarbe B., Le Guerneve C., Cheynier V., Moutounet M. (2000). Phenolic composition of grape stems. J. Agric. Food Chem..

[31] Downey M.O., Mazza M., Krstic M.P. (2007). Development of a stable extract for anthocyanins and flavonols from grape skin. Am. J. Enol. Vitic..

[32] Liang N.N., He F., Pan Q.H., Wang J., Reeves M.J., Duan C.Q. (2012). Optimization of sample preparation and phloroglucinol analysis of Marselan grape skin proanthocyanidins using HPLC-DAD-ESI- MS/MS. S. Afr. J. Enol. Vitic..

[33] He J.J., Liu Y.X., Pan Q.H., Cui X.Y., Duan C.Q. (2010). Different anthocyanin profiles of the skin and the pulp of Yan73 (Muscat Hamburg × Alicante Bouschet) grape berries. Molecules.

[34] Li Q., He F., Zhu B.Q., Liu B., Sun R.Z., Duan C.Q., Reeves M.J., Wang J. (2014). Comparison of distinct transcriptional expression patterns of flavonoid biosynthesis in Cabernet Sauvignon grapes from east and west China. Plant Physiol. Biochem..

[35] Wang Y., Chen W.K., Gao X.T., He L., Yang X.H., He F., Duan C.Q., Wang J. (2019). Rootstock-mediated effects on Cabernet Sauvignon performance: Vine growth, berry ripening, flavonoids, and aromatic profiles. Int. J. Mol. Sci..

[36] Siren J., Valimaki N., Makinen V. (2014). Indexing graphs for path queries with applications in genome research. IEEE ACM Trans. Comput. Biol. Bioinform..

[37] Trapnell C., Williams B.A., Pertea G., Mortazavi A., Kwan G., van Baren M.J., Salzberg S.L., Wold B.J., Pachter L. (2010). Transcript assembly and quantification by RNA-Seq reveals unannotated transcripts and isoform switching during cell differentiation. Nat. Biotechnol..

[38] Sun R.Z., Pan Q.H., Duan C.Q., Wang J. (2015). Light response and potential interacting proteins of a grape flavonoid 3′-hydroxylase gene promoter. Plant Physiol. Biochem..

[39] Ruijter J.M., Ramakers C., Hoogaars W.M.H., Karlen Y., Bakker O., van den Hoff M.J.B., Moorman A.F.M. (2009). Amplification efficiency: Linking baseline and bias in the analysis of quantitative PCR data. Nucleic Acids Res..

[40] Clough S.J. (2010). Floral dip: A simplified method for Agrobacterium-mediated transformation of *Arabidopsis thaliana*. Plant J..

[41] Jefferson R.A., Kavanagh T.A., Bevan M.W. (1987). GUS fusions: β-glucuronidase as a sensitive and versatile gene fusion marker in higher-plants. Embo J..

[42] Tanabe N., Tamoi M., Shigeoka S. (2015). The sweet potato RbcS gene (IbRbcS1) promoter confers high-level and green tissue-specific expression of the GUS reporter gene in transgenic Arabidopsis. Gene.

[43] Thompson R.S., Jacques D., Haslam E. (1972). Plant proanthocyanidins—Part 1. Introduction; the isolation, structure, and distribution in nature of plant procyanidins. J. Chem. Soc. Perk. Trans..

[44] He F., Pan Q.H., Shi Y., Duan C.Q. (2008). Biosynthesis and genetic regulation of proanthocyanidins in plants. Molecules.

[45] Bogs J., Ebadi A., McDavid D., Robinson S.P. (2006). Identification of the flavonoid hydroxylases from grapevine and their regulation during fruit development. Plant Physiol..

[46] Fujino N., Tenma N., Waki T., Ito K., Komatsuzaki Y., Sugiyama K., Yamazaki T., Yoshida S., Hatayama M., Yamashita S. (2018). Physical interactions among flavonoid enzymes in snapdragon and torenia reveal the diversity in the flavonoid metabolon organization of different plant species. Plant J..

[47] Winkel-Shirley B. (1999). Evidence for enzyme complexes in the phenylpropanoid and flavonoid pathways. Physiol. Plant.

[48] Jaakola L. (2013). New insights into the regulation of anthocyanin biosynthesis in fruits. Trends Plant Sci..

[49] Matus J.T., Poupin M.J., Canon P., Bordeu E., Alcalde J.A., Arce-Johnson P. (2010). Isolation of WDR and bHLH genes related to flavonoid synthesis in grapevine (*Vitis vinifera* L.). Plant Mol. Biol..

[50] Fujita A., Soma N., Goto-Yamamoto N., Mizuno A., Kiso K., Hashizume K. (2007). Effect of shading on proanthocyanidin biosynthesis in the grape berry. J. Jpn. Soc. Hortic. Sci..

[51] Fujita A., Goto-Yamamoto N., Aramaki I., Hashizume K. (2006). Organ-specific transcription of putative flavonol synthase genes of grapevine and effects of plant hormones and shading on flavonol biosynthesis in grape berry skins. Biosci. Biotechnol. Biochem..

[52] Martens S., Teeri T., Forkmann G. (2002). Heterologous expression of dihydroflavonol 4-reductases from various plants. Febs Lett..

[53] Kolb C.A., Kopecky J., Riederer M., Pfundel E.E. (2003). UV screening by phenolics in berries of grapevine (*Vitis vinifera*). Funct. Plant Biol..

[54] Bakowska A., Kucharska A.Z., Oszmianski J. (2003). The effects of heating, UV irradiation, and storage on stability of the anthocyanin-polyphenol copigment complex. Food Chem..

[55] Calderon A.A., Garciaflorenciano E., Munoz R., Barcelo A.R. (1992). Gamay grapevine peroxidase—Its role in vacuolar anthocyani(di)n degradation. Vitis.

[56] Biruk A., Asfaw D., Manela N., Perl A., Oren-Shamir M., Fait A. (2015). Metabolite profiling and transcript analysis reveal specificities in the response of a berry derived cell culture to abiotic stresses. Front. Plant. Sci..

[57] Koyama K., Goto-Yamamoto N. (2008). Bunch shading during different developmental stages affects the phenolic biosynthesis in berry skins of ‘Cabernet Sauvignon’ grapes. J. Am. Soc. Hortic. Sci..

[58] Diharce J., Golebiowski J., Fiorucci S., Antonczak S. (2016). Fine-tuning of microsolvation and hydrogen bond interaction regulates substrate channelling in the course of flavonoid biosynthesis. Phys. Chem. Chem. Phys..

[59] Terzaghi W.B., Cashmore A.R. (1995). Light-regulated transcription. Annu. Rev. Plant. Phys..

[60] Hahn S., Buratowski S., Sharp P.A., Guarente L. (1989). Yeast TATA-binding protein TFIIIB binds to TATA elements with both consensus and nonconsensus DNA-sequences. Proc. Natl. Acad. Sci. USA.

[61] Mazarei M., Ying Z., Houtz R.L. (1998). Functional analysis of the Rubisco large subunit N-epsilon-methyltransferase promoter from tobacco and its regulation by light in soybean hairy roots. Plant. Cell Rep..

