# Supplementary material for: The Effect of Light Intensity on the Expression of Leucoanthocyanidin Reductase in Grapevine Calluses and Analysis of Its Promoter Activity
Source: Genes (Basel). 2020 Sep 30;11(10):1156. doi: 10.3390/genes11101156 (PMC7600843; doi:10.3390/genes11101156)
Supplement: Supplementary file 1 [file genes-11-01156-s001.zip › supplementary/Supplementary figures.docx]

**
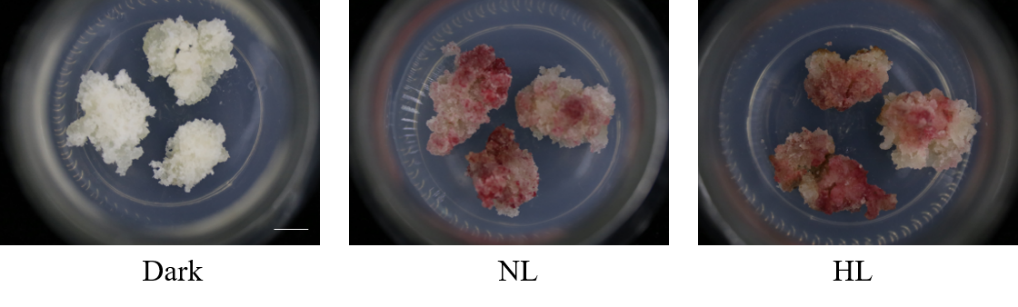
**

**Figure S1.** Phenotypes of grapevine calluses treated with different light intensities. NL, control condition; HL, high light. Bar = 10 mm.


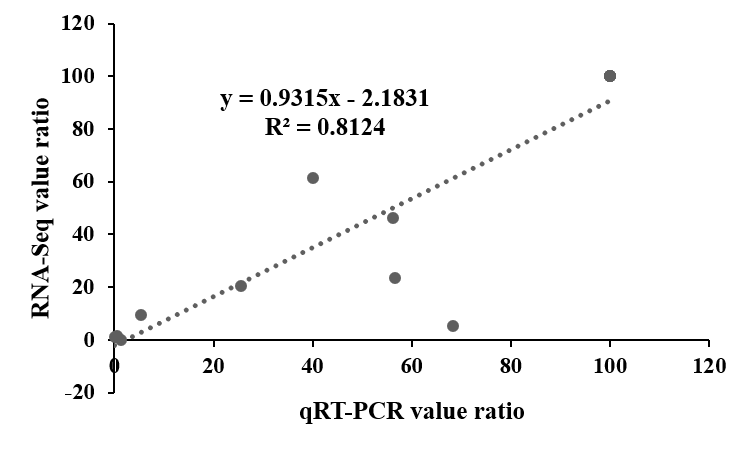


**Figure S2.** Comparison of gene expression determined by RNA-Seq and by qRT-PCR. Data were from six genes across three different light intensity treatments, *VviUbiquitin1* was used as the reference gene in qRT-PCR. The RNA-Seq values (normalized) were plotted against the qRT-PCR values (normalized). The normalized values were determined by calculating the relative expression ratio value for each treatment relative to the highest value for each gene. Linear regression analysis gave an overall coefficient of variation of 0.8124.

**
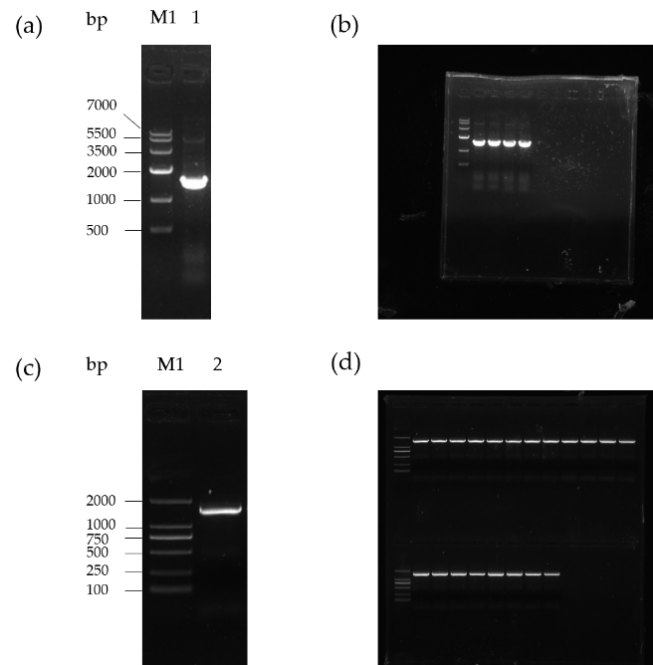
**

**Figure S3.** PCR products of p*VviLAR1* and p*VviLAR2* analyzed with 1.5% agarose gel electrophoresis. (a) Lane 1: p*VviLAR1*, lane M1: DNA marker Ⅳ (TIANGEN, China); (b) The original gel of the agarose gel in **Figure S3 (a)**; (c) Lane 2: p*VviLAR2*, lane M2: DNA marker D2000 (TIANGEN, China); (d) The original gel of the agarose gel in **Figure S3 (c)**.


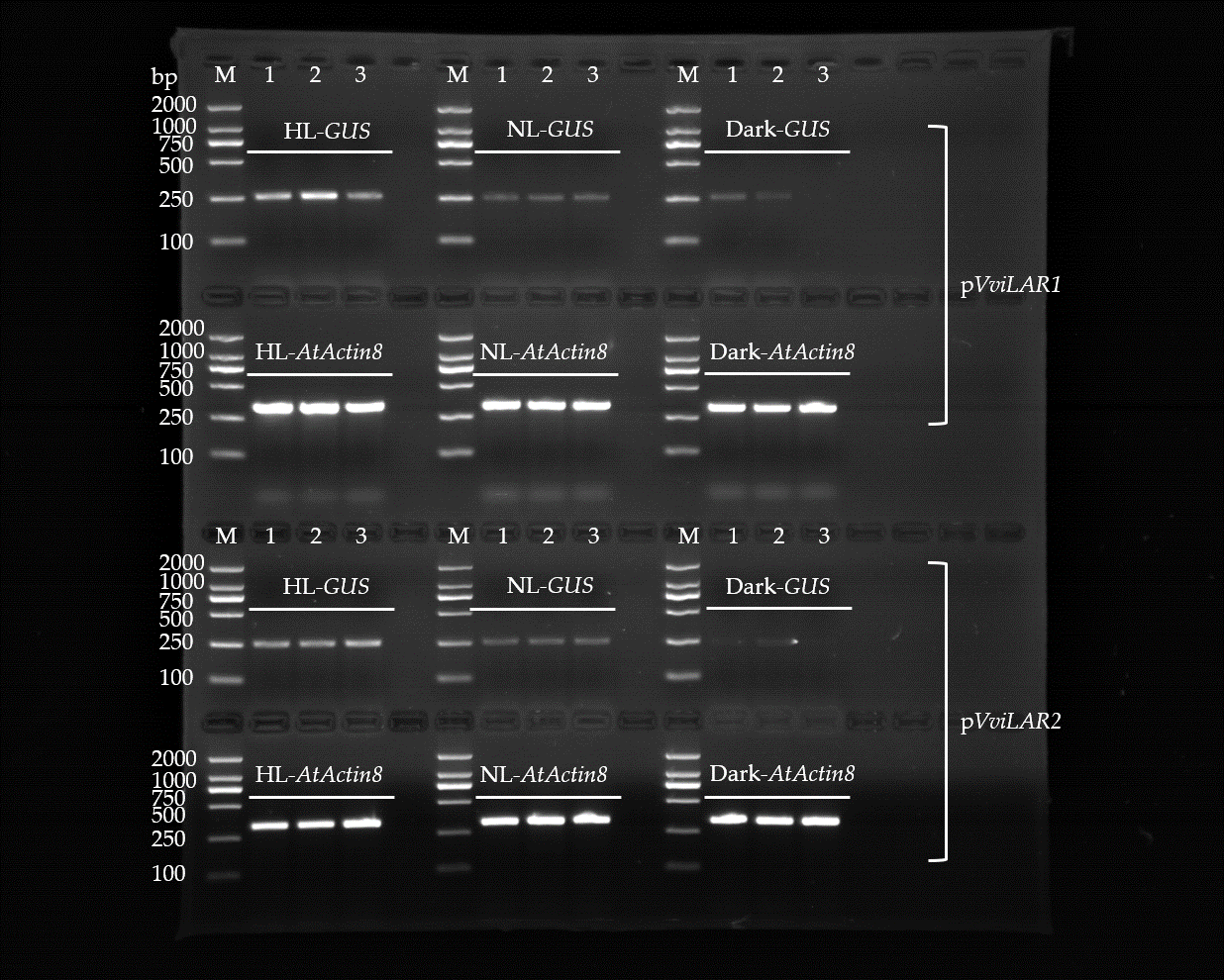


**Figure S4.** The original gel of *GUS* and *AtActin8* expression detection in **Figure 6**. M: DNA marker D2000 (TIANGEN, China); HL: high light; NL: control condition. The number ‘1-3’ mean the different lines used for the light intensity treatments, which were corresponded to the left, middle, and right leaf of p*VviLAR1* or p*VviLAR2* in each light intensity treatment in **Figure 6**, respectively.
